# Circumferential full-thickness skin grafting: An excellent method for the treatment of short penile skin in adult men

**DOI:** 10.3389/fsurg.2022.999916

**Published:** 2022-10-24

**Authors:** Kexin Che, Keke Wang, Ye Yuan, Fengyong Li, Qiang Li

**Affiliations:** Plastic Surgery Hospital, Chinese Academy of Medical Sciences and Peking Union Medical College, Beijing, China

**Keywords:** full-thickness skin grafting, penile skin, scrotum, retrospective analysis, outcomes

## Abstract

**Objective:**

Short penile skin due to excessive circumcision is a complex condition requiring surgical care. The study aims to investigate the effect of full-thickness skin grafting (FTSG) in treating short penile skin.

**Methods:**

A retrospective analysis was performed on 24 patients with insufficient penile skin. The included patients underwent full-thickness skin grafting from the scrotum and the groin region in the Genital Plastic Surgery Center between February 2014 and September 2021. Morphology of the penis, length of the penis, complication, and donor area healing status was observed by the authors. Additionally, the International Index of Erectile Function Questionnaire (IIEF-5) and the patient's evaluation of penile appearance were investigated.

**Results:**

An aesthetically pleasing appearance of the penis was obtained by FTSG in 24 patients. The length of penis was improved after surgery (5.70 cm ± 1.24 cm vs. 6.05 cm ± 1.33 cm, *P* value < 0.05). All patients had good healing of the penile area without serious complications. Only 2 cases showed minor partial necrosis and recovered soon after proper treatment. A high patient's evaluation of penile appearance was received (4.08 ± 0.71, mean ± SD) and the scores of IIEF-5 increased significantly after surgery (18.38 ± 2.24 vs. 21.08 ± 1.79, *P*-value < 0.05).

**Conclusions:**

FTSG from the scrotum and inguinal skin provides good aesthetic and functional results for treating short penile skin. FTSG, particularly the scrotum-derived skin graft, offers a great supplement to penile skin. It could be suggested as a promising method of treating insufficient penile skin, which meets both functional and aesthetic needs.

## Introduction

The normal appearance of male external genitalia is important for self-esteem and sexuality. The surface of the penis body is covered with loose skin (1). Globally, 37%–39% of men undergo circumcision for sociocultural and personal preferences, penile lesion excision, and the prevention of sexually transmitted diseases (2). When an adult is circumcised, there can be a range of symptoms if too much skin is removed. Common symptoms include pain due to skin tightness, discomfort during sex activities, premature ejaculation, restricted erection, and hypersensitive penis (3, 4).

Short penile skin due to excessive circumcision is a complex condition requiring surgical care (5, 6). It is actually a functional loss of penile skin. Nevertheless, reconstruction of penile skin is challenging due to its special anatomy and functional requirements such as sliding. According to previous literature, split-thickness skin graft (STSG) and pedicled scrotal flaps were used for treating penile defects (1, 7). Both approaches, however, do not appear to meet the high demand for penile shape and sexual function in patients with short penile skins. We, therefore, tried to explore better methods that address both functional and cosmetic requirements. Herein, a retrospective study is designed to investigate the effect of full-thickness skin grafting (FTSG) in treating short penile skin.

## Methods

### Patients

The study included all patients with insufficient penile skin who underwent FTSG surgery at Genital Plastic Surgery Center between February 2014 and September 2021. A retrospective study was conducted based on clinical data, operation records, and clinical photography. The mean age of the patients was 45.33 ± 4.95 years (mean ± SD, Table 2). Each patient had previous excessive circumcisions of the prepuce. The common symptoms were discomfort and pain in the penis, tight penile skin, penile hypersensitivity, and premature ejaculation. The penis length of each patient was measured in the unstretched and unerectile condition before surgery and during follow-up. FTSG from the scrotum was carried out in twenty-one patients. The groin area was selected as the donor region in three patients because the scrotum had hair, lesions, or an operation history. During the follow-up, the appearance of the penis and donor areas was photographed and recorded. The erectile function was evaluated through scores of the International Index of Erectile Function Questionnaire (IIEF-5) both before and after the surgery. The IIEF-5 is an abridged five-item version of the 15-item International Index of Erectile Function to evaluate the presence and severity of erectile dysfunction (8). In addition, patients were asked if they had abnormalities in the urination process at each follow-up and the relevant results were summarized by the authors. All patients scored the appearance of the penis after the surgery non-anonymously on a scale of 5 – “extremely satisfied with the appearance of the penis”, 4 – “very satisfied with the appearance of the penis”, 3 – “moderately satisfied with the appearance of the penis”, 2 – “a little satisfied with the appearance of the penis”, and 1 – “not at all satisfied with the appearance of the penis”.

### Surgical procedures

**Figure 1:**
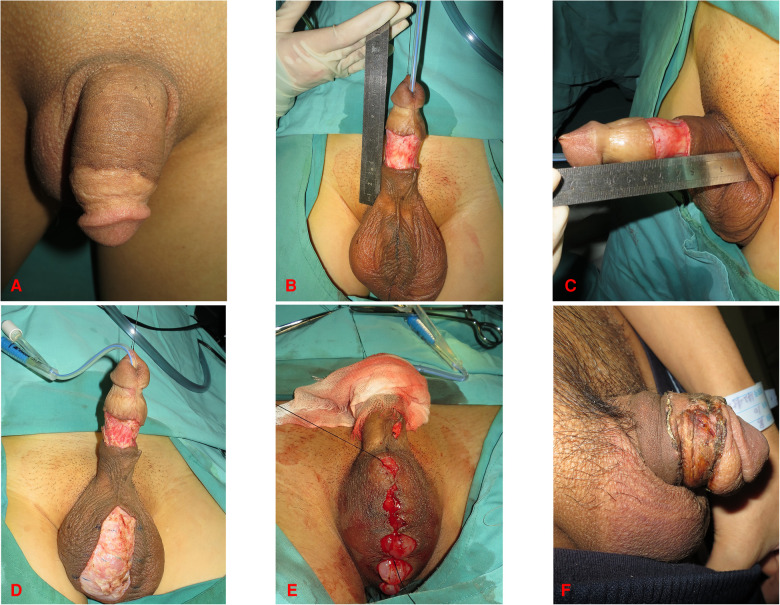
42-year-old male. (**A**) Preoperative photograph of the penis. (**B,C**) Incision of the penile skin. (**D**) Scrotal wound after skin removal. (**E**) Closure of the scrotal incision. (**F**) Morphology of the penis three weeks after surgery.

**Figure 2:**
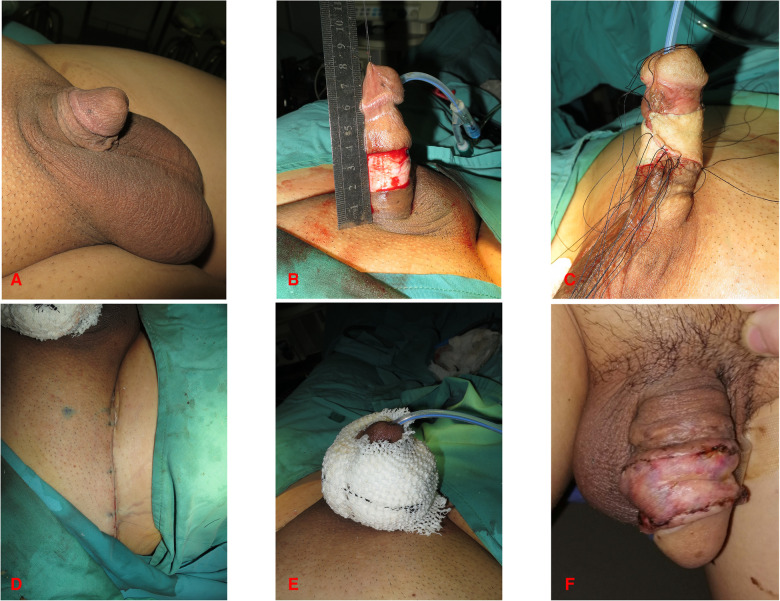
42-year-old male. (**A,B**) Preoperative photograph of the penis. (**C**) Incision of the penile skin. (**D,E**) Circumferential full-thickness skin grafting. (**D**) Sutured inguinal area incision. (**E**) Pressure dressing of penis. (**F**) Follow-up at one month after surgery.

**Figure 3:**
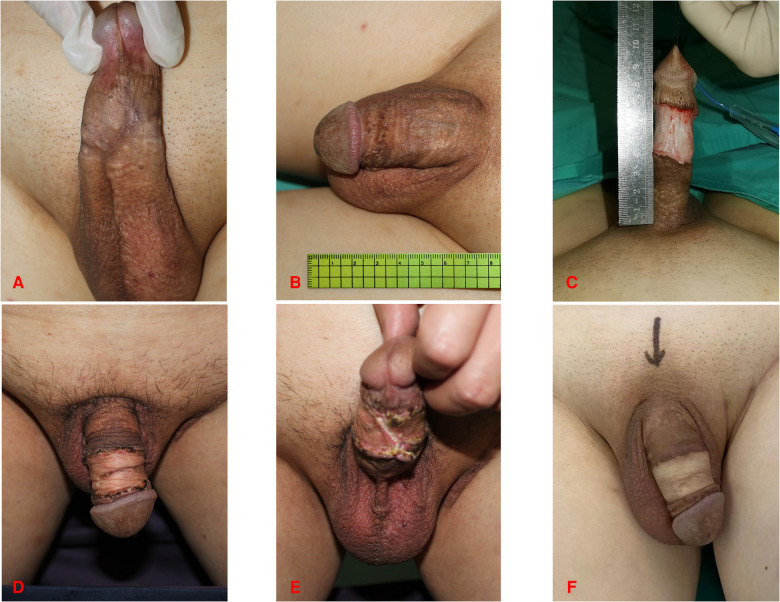
47-year-old male. (**A,B**) Preoperative photograph of the penis. (**C**) Incision of the penile skin. (**D,E**) Three months after penial skin grafting from the inguinal region. (**F**) Follow-up at one year after surgery.

**Figure 4:**
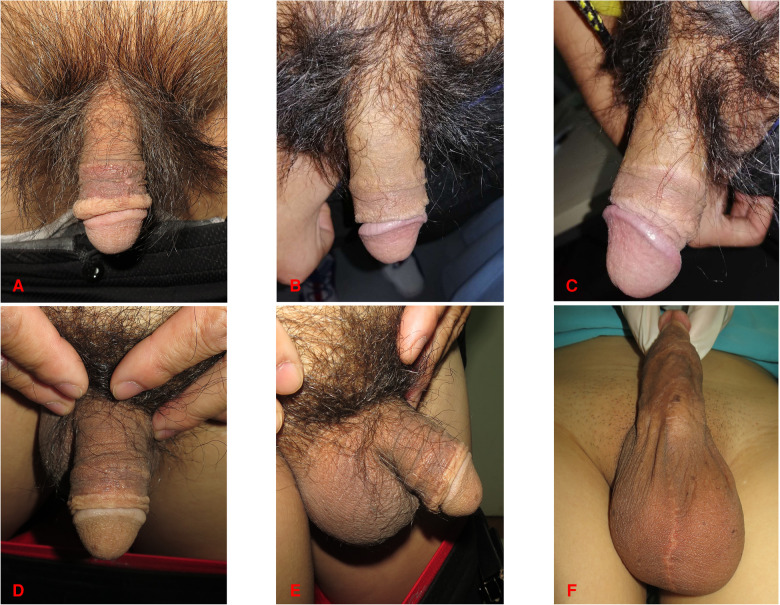
Same patient as in Figure 1. (**A**) Three months after surgery. (**B,C**) Six months after surgery. (**D**) Photograph of the dorsal side of the penis 12 months after surgery. (**E**) Photograph of the lateral side of the penis 12 months after surgery. (**F**) Healing status of the donor site 12 months after the surgery.

The surgeries were carried out under a general anesthetic and patients were placed in a supine position. Place the double-lumen urethral catheter at the beginning of the procedure. The skin of the penis was incised cautiously to reserve the subcutaneous fascia (Figures 1B,C, 2B, 3C). The proximal skin shrank back toward the penile root about 2 cm–3 cm after releasing the adherent tissue. Adequate hemostasis of the exposed wound was applied by electrocautery. The penis should be fully stretched to imitate the erectile state. The exposed penile wound was measured by the surgeons (Figures 1, 3, 4). The skin donor site in twenty-one patients was the scrotum, and inguinal skin was chosen in three. For skin harvesting on the groin, the inguinal crease was used as the long axis and for the skin harvesting on the scrotum, the midline of the scrotum was chosen as the incision. The FTSG was designed to be 10%–20% larger than the surface of the wound. Following the marking of the graft outlines, we made an incision with a 45° angle inside along a boundary of the constructed skin graft and harvested it by slicing it. The obtained skin was trimmed into FTSGs with sharp scissors (Figure 2C). Then, the FTSG was wrapped around the penis. It was sewn into the surrounding penile skin with 3–0 nonabsorbable braided sutures. During intermittent suturing, excess sutures were not trimmed to prepare for pressure dressing. The oiled gauze and plain gauze were wrapped surrounding the grafted skin pieces layer by layer like the shape of a “doughnut” (Figure 2E). The retained sutures were cross-knotted with thick wound dressings. The pressure dressing was placed for at least ten days. When closing the donor area, the inguinal area was treated with 3–0 and 4–0 absorbable sutures layer by layer, and the skin was closed with 5–0 nonabsorbable monofilaments. For the scrotal donor area, it was closed with 3–0 absorbable sutures subcutaneously and the skin was interrupted sutured with 5–0 nonabsorbable monofilaments. At the end of the surgical procedure, check the color and texture of the exposed glans.

## Results

All included patients received aesthetic and well-functioning penis under FSTG. The IIEF-5 scores were significantly improved after surgery (18.38 ± 2.24 vs. 21.08 ± 1.79, *P*-value < 0.05, Table 1). The patients' evaluation of penis morphology was 4.08 ± 0.71 (mean ± SD) based on a 5-points scale (Tables 1, 2). The average operating time was 4.06 ± 0.28 h (Table 2). The pre-operative penis length was 5.70 cm ± 1.24 cm (mean ± SD) and the post-operative one was 6.05 cm ± 1.33 cm (mean ± SD). There was a significant difference between the two groups (*P* value < 0.05, Table 2). The follow-up was 4–18 months (mean ± SD, 8.79 ± 0.71, Table 1). The FTSG on the penis survived well in all patients without major complications such as severe infection, wound dehiscence, bloated appearance, glans edema, obvious scars, or deformity. Only two patients had minor partial necrosis and soon recovered under disinfection and dressing change of wounds. All patients had satisfactory healing of the donor area without wound dehiscence, infection, obvious scars, or deformity. Based on the post-operative photographs, it was found that FTSG from the scrotum showed a more similar color to the recipient site of the penis than the groin area-derived skin grafts. During the follow-up period, no patient reported an abnormal urination process (Table 1).

**Table 1:** Postoperative follow-up of the included patients.

Serial number	Follow-up (month)	Penis length (cm)		IIEF-5 scores		Penile appearance scores provided by the patients	Reported abnormal urination from patients	Complications
		Preoperative	Postoperative	Preoperative	Postoperative			
1	4	7.1	7.5	20	20	4	None	None
2	4	6.5	7.4	16	19	5	None	None
3	18	7.1	7.3	17	23	4	None	None
4	9	4.5	4.6	19	19	3	None	None
5	8	7.1	7.9	20	23	5	None	None
6	15	6.7	7.2	20	23	5	None	None
7	15	7.5	7.8	17	23	5	None	None
8	12	4.2	4.5	16	22	4	None	Minor partial necrosis
9	4	4.3	4.6	19	19	3	None	None
10	4	4.7	5	18	20	5	None	None
11	4	4.5	4.8	16	21	4	None	None
12	6	7.7	7.9	18	22	4	None	None
13	8	5.3	5.3	17	22	5	None	None
14	8	4.2	4.4	21	22	3	None	None
15	9	4.5	5.1	14	23	4	None	None
16	18	6.3	6.5	16	23	5	None	Minor partial necrosis
17	6	6.2	6.2	18	18	3	None	None
18	6	5.5	5.8	21	21	4	None	None
19	12	4.7	5.2	23	22	4	None	None
20	18	4.5	4.8	21	22	4	None	None
21	10	7.2	8.1	22	22	4	None	None
22	4	5.4	5.5	17	19	3	None	None
23	4	4.3	4.6	18	21	5	None	None
24	5	6.8	7.1	17	17	3	None	None
Mean ± SD	8.79 ± 4.91	5.70 ± 1.24	6.05 ± 1.33	18.38 ± 2.24	21.08 ± 1.79	4.08 ± 0.71		

IIEF-5: the international index of erectile function questionnaire. Score range: 5–25.

**Table 2:** Clinical data of the 24 included patients.

	Mean ± SD	Range
Patient age	45.33 ± 4.95	(38–58)	* *
Follow-up	8.79 ± 0.71	(4–18)	*P* value < 0.05
Pre-operative penis length (cm)	5.70 ± 1.24	(4.2–7.7)	
Post-operative penis length (cm)	6.05 ± 1.33	(4.4–8.1)	* *
Operative time (hour)	4.06 ± 0.28	(3.2–5.1)	* *
Patient's scoring of penile appearance	4.08 ± 0.71	(3–5)	*P* value < 0.05
Pre-operative score of IIEF-5	18.38 ± 2.24	(14–23)	
Pre-operative score of IIEF-5	21.08 ± 1.79	(17–23)	

Ratings of penile appearance during follow-ups: 5 – “extremely satisfied with the appearance of the penis”, 4 – “very satisfied with the appearance of the penis”, 3 – “moderately satisfied with the appearance of the penis”, 2 – “a little satisfied with the appearance of the penis”, and 1 – “not at all satisfied with the appearance of the penis”.

IIEF-5: the international index of erectile function questionnaire. Score range: 5–25.

## Discussion

Excessive circumcision is a common reason for short penile skin, which leads to functional and aesthetic problems, such as the shortening of penis skin, iatrogenic hidden penis, hyperesthesia, chronic pain, and ulcer-prone scars (5, 9). Conditions due to excessive circumcision, such as erectile limitations, and concealed and sensitive penis, require further treatment (2, 7). In this study, all patients had a history of circumcision, and patients reported that after the procedure they experienced discomforts such as penile pain, tight penile skin, hypersensitivity, and premature ejaculation. The primary purpose of treating penile skin deficiency is to supplement the skin of the penis and relieve associated discomfort. The procedure has to be simple, reliable, tensionless, and aesthetically pleasing.

All patients received aesthetic and functional penises *via* FTSG based on the authors' observation of photographs. A high patient's evaluation of the penis morphology was received based on a 5-points scale and the length of the penis increased significantly after surgery (Tables 1, 2). The aim of FTSG is not only to replenish penile skin but also to improve the quality of sex activities. To evaluate the presence and severity of erectile dysfunction, the questionnaire of IIEF-5 was provided to the patients before the surgery and during the follow-up. It was found that the IIEF-5 scores of patients were significantly improved after surgery (*P*-value < 0.05, Tables 1, 2).

During the surgical procedure, FTSG was defatted and trimmed to a thin skin sheet to maximize the engraftment potential. The penile skin wound was covered with FTSG from the scrotum in twenty-one patients. Patients who received scrotal skin grafts developed hyperpigmentation in the short term, but the grafted skin turned more aesthetically pleasing over time (Figures 1, 4). Similarities were reported in the aspects of thickness, laxity, and color between penile and scrotal skins (10). The skin of the scrotum is thin, soft, flexible, and expandable. It has penis-like properties, a rich blood supply, and little subcutaneous fat (11). The skin color of the scrotum is more similar to the original skin of the penis compared to the inguinal region, according to the author's observation. It is an ideal donor area for repairing penile defects. Direct suturing is tolerable if less than 50% of the scrotal skin is removed (12). For instance, it was reported that after removing 10 cm × 5 cm scrotal skin, the incision can still be directly pulled together and sutured (13). In three patients, the scrotum was unsuitable for the donor area (one patient had scrotal lesions, one had a hairy scrotum, and one had a history of scrotal surgery), therefore, the inguinal skin was chosen instead (Figure 2).

FTSG had several advantages for treating penile skin shortage, compared to STSG. FTSG has a softer texture, less contraction, better elasticity, and better abrasion resistance because it contains more dermis. Besides, it has better potential for sensory recovery (1, 14, 15). Over time, FTSG could resist the problem of contraction better, while STSG can lose up to 20% of its surface area from contraction (16–18). Thus, FTSG could be more conducive to meeting the demand for extra skin under the erectile states of the penis. Generally, STSG is thinner and has less tissue, less metabolic demand from the wound bed, and a better survival rate (19, 20). Notably, in this study, the survival rate of FTSG was 100%, and none of the 24 patients had non-healing or infectious wounds. Only two patients showed minor partial necrosis and recovered soon after proper treatment. Thus, the survival rate of FTSG during penile skin repair is reliable. For the replacement and repair of penile skin, pedicled scrotal flaps are reported in some literature (1, 21). However, after flap repair, the penis may become bulky with obvious deformity of the scrotum, which is not good for sexual activities (22). In this study, the included patients received aesthetic and well-functioning penises. The scars on the scrotum were not obvious (Figure 4F). Compared with scrotal flap repair, skin grafting has fewer surgical steps, shorter hospital stays, and less morbidity in the donor area (21, 23).

The patient's functional status and expectations should be well understood when treated with FTSG. The range of skin grafts should ensure that the erection of patients is not affected. The measurement of the skin grafts may not be accurate unless an erection was induced or the penis was in a fully stretched state. Considering the unique functional requirements and contraction of grafted skin, it requires 10%–20% extra skin to ensure skin sliding (24). The integrity of the subcutaneous fascia was preserved during circumferential incision to maintain the good sliding of the penile skin. None of the patients had postoperative glans edema under sufficient skin grafting in this study.

Several factors will lead to failure of FTSG, such as fluid accumulation, infection, and grafted skin pieces dislocation (1, 25). Typically, the process of neovascularization can be advanced as long as the grafted skin clings to the recipient bed for 3–5 days without movement (26). The movement and dislocation of skin grafts could disrupt the neovascularization from the recipient bed to the graft (27). Reliable dressing of the penis is vital for maintaining the shape of the penis and the survival of skin grafts (18).

When stitching up the grafted skin, we chose to reserve the stitches at a certain length and applied compression dressings around the circumference of the penis, like a “doughnut” (Figure 2E). The circumferential compression dressing should be done while the penis is in traction to maintain the shape as much as possible.

There are some shortcomings in the study that cannot be ignored. For instance, there are few objective assessments for the evaluation of the penis. Scoring from patients and photographs during follow-up are not sufficient for a solid methodology. Also, only 24 patients were included in the single-center retrospective study. The data presented here might be limited, and caution is warranted when interpreting the findings. Moreover, the follow-up period was 4–18 months and 17 patients (70.83%) were less than one year. The long-term effect of FTSG was not explored in the study, thus, increasing the follow-up is essential to get a more reliable result. More large-scale studies from multi-centers and prospective studies are needed to further validate our results in the future.

## Conclusions

FTSG can provide an aesthetically pleasing appearance to the penis and improve erectile function. When treating the penile skin shortage, FTSG from the scrotum and inguinal skin is a safe, effective, and aesthetically pleasing choice. It offers a good combination of cosmetic and functional results without donor site morbidity.

## Data Availability

The original contributions presented in the study are included in the article/Supplementary Material, further inquiries can be directed to the corresponding author/s.
